# Magnetic resonance imaging diagnosis of Mayer-Rokitansky-Kuster-Hauser syndrome

**DOI:** 10.4103/0974-1208.44117

**Published:** 2008

**Authors:** MJ Govindarajan, Revathi S Rajan, Arjun Kalyanpur, Ravikumar

**Affiliations:** Teleradiology Solutions, Bangalore, India; 1 Bangalore Assisted Conception Centre, Bangalore, India

**Keywords:** Mullerian agenesis, MRI, MRKH syndrome

## Abstract

Magnetic resonance imaging (MRI) is the mainstay in the imaging evaluation of Mullerian agenesis, but is not routinely being utilized, particularly in India. Though sagittal MRI clearly demonstrates the absence or hypoplasia of the uterus and the axial images demonstrate the normal ovaries, it is the ability to identify and objectively evaluate other associated anomalies that makes MRI a unique diagnostic modality. It is also noninvasive and has multiplanar capabilities at the same time having a very high soft tissue resolution. We presume it can be used as a comprehensive imaging package for evaluating these patients at one sitting. We report a case of Mullarian agenesis presenting as primary amenorrhea stressing the role and benefits of MRI.

## INTRODUCTION

Mayer- Rokitansky-Kuster-Hauser (MRKH) syndrome is an uncommon variation in the prenatal development of the female genital tract. It is a congenital malformation of the female genital tract. Its features include partial or complete absence (agenesis) of the uterus with an absent or hypoplastic vagina normal fallopian tubes, ovaries, normal external genitalia and the typical 46, XX, female chromosome pattern. Breast development and growth of pubic hair are also normal. Associated renal and/or skeletal abnormalities are common. Mayer-Rokitansky-Kuster-Hauser syndrome is also known as Mullerian Agenesis. The incidence is one in 4000–5000 female newborns. Mayer—Rokitansky—Kuster—Hauser (MRKH) syndrome is a partial or complete absence (agenesis) of the uterus with an absent or hypoplastic vagina. The aetiology is thought to be polygenic multi-factorial; occasionally, the syndrome results from a genetic mutation or deletion of genes on chromosome 16. The normal external appearance of MRKH females makes it difficult to diagnose until puberty, typically diagnosed in mid-adolescence. The average age of diagnosis is between 15 and 18 years, although occasionally a girl may be diagnosed at birth or during childhood because of other health problems. A pelvic ultrasound may be used to see the presence or absence of the uterus and its condition.

## CASE REPORT

**Figure 1:**
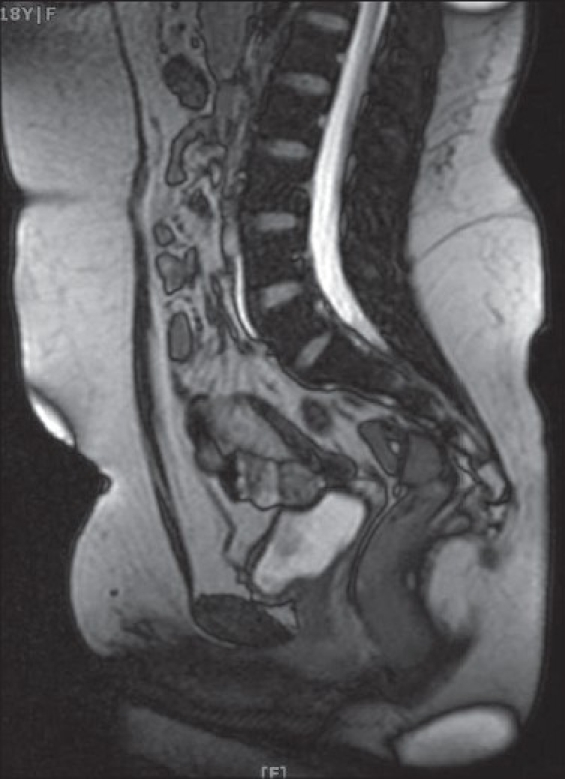
Sagittal T2W image in mid sagittal palne. No uterus can be made out and only the lower vagina is seen

**Figure 2:**
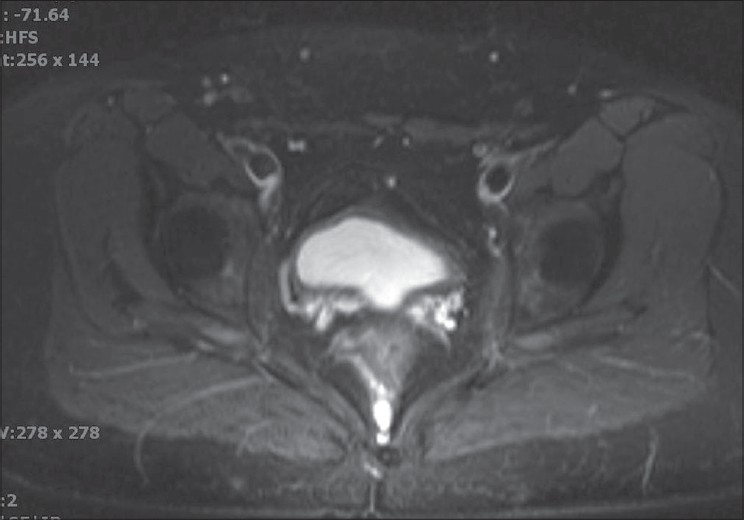
Axial T2W image at the level acetabulum showing absent vagina between the bladder and rectum

**Figure 3:**
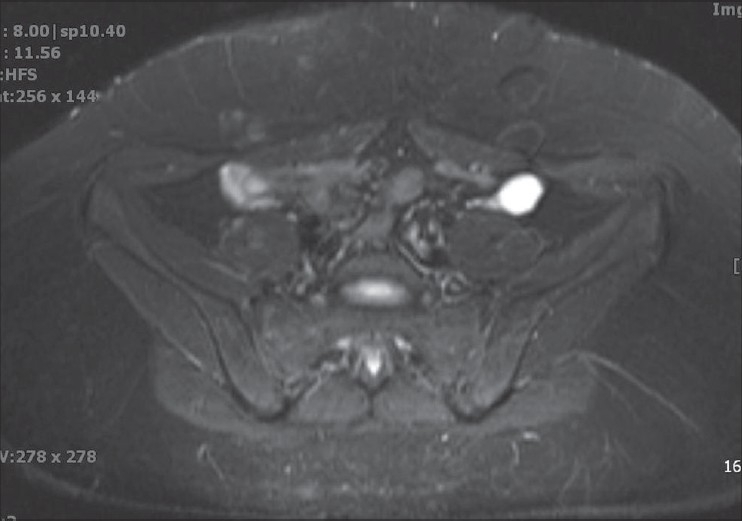
Axial T2W image at a higher level demonstrates normal ovaries with follicles

An 18-year-old female presented with primary amenorrhea. On examination, the patient's secondary sexual characteristics were found to be normal. An MRI of the pelvis was performed [Figures 1–3]. The sagittal T2 W MRI [Figure 1] demonstrated absence of uterus and upper vagina. The axial T2 FS images [Figures 2 and 3] confirmed the presence of normal ovaries with follicles and absence of vagina between the rectum and bladder. The visualized portions of the kidneys were unremarkable. No other abnormalities were identified.

## DISCUSSION

Uterine malformations occur in about 0.1–0.5% of all women.[1] Mayer-Rokitansky-Kuster-Hauser (MRKH) syndrome is a rare developmental failure of a part or whole of the Mullerian duct resulting in congenital absence of uterus and vagina with a prevalence of 1 in 4000–5000 female births. It accounts for approximately 15% of patients with primary amenorrhea and is also the second commonest cause.

Patients with MRKH syndrome have 46 XX as karyotype. The secondary sexual characteristics are normal as the ovaries function normally. The external genitalia appear normal, although in reality, a shallow vaginal pouch may be present.

The syndrome was first described by Mayer in 1829. Initial description consisted of various vaginal anomalies like duplications due to abnormal development of the Mullerian ducts. Later in 1838, Rokitansky described uterine and vaginal agenesis. Kuster recognized renal abnormalities, such as renal ectopy or agenesis as well as skeletal abnormalities in 1910. Other rare associations are cardiac anomalies and anorectal malformations (ARM). Hauser distinguished MRKH from testicular feminization in 1961.

The diagnosis is frequently made clinically, but often confirmed either radiologically or laparoscopically in patients whose hormonal and karyotypic investigations for primary amenorrhea are normal. Two-dimensional ultrasound is the initial choice of diagnostic modality, but three-dimensional ultrasound maybe more sensitive. When ultrasound is equivocal, computed tomography (CT) can detect and differentiate congenital anomalies, but it is not routinely performed due to ionizing radiations. An MRI can be more effective owing to its multiplanar capability and the best soft tissue contrast compared to any other imaging modality, without the use of ionizing radiations.[1]

Uterine agenesis or hypoplasia is best diagnosed on T2 weighted sagittal images. The slice thickness should be 5 mm or less. Uterine hypoplasia may be diagnosed when there is small uterus and reduced intercornual distance (< 2 cm), but the patients may also have poor zonal differentiation and reduced endometrial and myometrial widths.[1,8] The endometrial cavity and the myometrium may be reduced in size. An endometrial segment may demonstrate increased signal intensity and be expanded depending on the presence of obstruction. An MRI has the ability to differentiate normal and abnormal uterus due to its exquisite soft tissue contrast resolution. Vaginal agenesis is best characterized on axial planes with no normal vaginal anatomy identified between the rectum and urethra. Its multiplanar capabilities are useful in the overall evaluation of the female pelvis, particularly when complex anorectal anomalies are expected. The normal ovaries can be well demonstrated with MRI where normal follicles can be identified. Normal ovaries are the major factors in the diagnosis of MRKH syndrome. The coronal MRI also helps to identify any associated renal malformations.

The clinical findings of MRKH syndrome are remarkable and a clinical diagnosis can be easily established. However, confirmation of the diagnosis, evaluating for other associated anomalies and sometimes to rule out a coexistent Turner's syndrome need further investigations including laparoscopy, imaging and karyotyping.

The classical case of MRKH syndrome, where the vagina is completely absent from the introitus, accounts for nearly 95% of all cases. The clinical diagnosis and surgical planning may relatively be simple. However, in the remaining 5% of patients, a blind upper one-third of the vagina can be present which cannot be satisfactorily evaluated by laparoscopy or ultrasound (2D or 3D). Our patient had a similar appearance. An MRI can definitely be more accurate and comprehensive in the evaluation of such patients. The congenital anomalies of the upper renal tracts can be associated in as many as 30–40% of the patients. The common types of renal anomalies may include renal agenesis and ectopic pelvic kidney.[9–12] When a coexistent renal anomaly is present, particularly an ectopic kidney in the pelvis or a horseshoe kidney where it is usually low placed; a laparoscopy may not be able to evaluate the abnormal position of the kidney, which is of significance during surgical management like vaginoplasty. An ultrasound examination will be able to identify these patients consistently, although, sometimes it may be difficult to visualize if bowel loops obscure the kidney or if the kidney is hypoplastic/aplastic. A CT scan is effective in evaluating such patients but at the cost of exposing the patient to ionizing radiations. When there are associated anorectal anomalies, an MRI can be invaluable in comparison with any other modality of investigation.

Currently, the most common pattern of management in a patient with Mullerian agenesis include investigation with clinical examination, karyotyping, ultrasound examination, intravenous pyelography (IVP) for the localization of kidneys and then laparoscopy in that order followed by vaginoplasty. The information obtained by ultrasound, IVP and diagnostic laparoscopy can all be obtained by the MRI alone – which is noninvasive, unlike laparoscopy and does not utilize radiations, unlike IVP.

Therefore, we conclude that MRI is the mainstay of imaging evaluation of MRKH syndrome, not only to confirm clinically diagnosed Mullerian anomalies of uterus but also to assess the degree of vaginal dysgenesis and associated anomalies like ARM and renal anomalies, which have an impact on the planning of treatment. With more sophisticated MR technology and availability of pelvic phase array coils, MRI is better equipped to evaluate these patients noninvasively. Though the cost and availability of MRI maybe a limiting factor, particularly in India, its very high soft tissue resolution, multiplanar capability and noninvasive but versatile nature makes MRI to be considered as a comprehensive package for the evaluation of these patients. We presume MRI can replace laparoscopy, particularly before planning surgery due to its noninvasive nature providing equally sufficient, if not more, information. An accurate diagnosis of MRKH is important as the patient can actually conceive and have their reproductive function fulfilled with the help of surrogate uterus.
